# Supplementary material for: Rapid Synthesis Method of Ag3PO4 as Reusable Photocatalytically Active Semiconductor
Source: Nanomaterials (Basel). 2022 Dec 24;13(1):89. doi: 10.3390/nano13010089 (PMC9823426; doi:10.3390/nano13010089)
Supplement: Supplementary file 1 [file nanomaterials-13-00089-s001.zip › nanomaterials-2099777-supplementary xml corrected-1.pdf]

nanomaterials

Supplementary Materials

Rapid Synthesis Method of Ag_3PO_4 as Reusable Photocatalytically Active Semiconductor

Zsejke-Réka Tóth [1,2,3,*], Diána Debreczeni [1], Tamás Gyulavári [1], István Székely [2,3], Milica Todea [2,4], Gábor Kovács [5], Monica Focşan [6], Klara Magyari [2], Lucian Baia [2,7,8], Zsolt Pap [1,2,8] and Klara Hernadi [1,9,*]

1 Department of Applied and Environmental Chemistry, Faculty of Science and Informatics, University of Szeged, Rerrich Béla sqr. 1, 6720 Szeged, Hungary;
2 Nanostructured Materials and Bio-Nano-Interfaces Center, Interdisciplinary Research Institute on Bio-Nano-Sciences, Babeș-Bolyai University, Treboniu Laurian str. 42, 400271 Cluj-Napoca, Romania;
3 Doctoral School in Physics, Faculty of Physics, Babes-Bolyai University, M. Kogălniceanu 1, 400084 Cluj-Napoca, Romania
4 Faculty of Medicine, Iuliu Hatieganu University of Medicine and Pharmacy, Victor Babeş 8, 400012 Cluj-Napoca, Romania
5 Department of Horticulture, Faculty of Technical and Human Sciences, Sapientia Hungarian University of Transylvania, Aleea Sighișoarei 1C, 530104 Târgu Mureș/Corunca, Romania;
6 Nanobiophotonics and Laser Microspectroscopy Center, Interdisciplinary Research Institute on Bio-Nano-Sciences, Treboniu Laurian str. 42, Babes-Bolyai University, 400271 Cluj-Napoca, Romania,
7 Faculty of Physics, Babeș-Bolyai University, M. Kogălniceanu str. 1, 400084 Cluj-Napoca, Romania
8 Institute of Research-Development-Innovation in Applied Natural Sciences, Babes-Bolyai University, Fântânele str. 30, 400294 Cluj-Napoca, Romania
9 Institute of Physical Metallurgy, Metal Forming and Nanotechnology; University of Miskolc, Miskolc-Egyetemváros, 3515 Miskolc, Hungary
* Correspondence: zsejke.toth@ubbcluj.ro (Z.-R.T.); klara.hernadi@uni-miskolc.hu (K.H.)

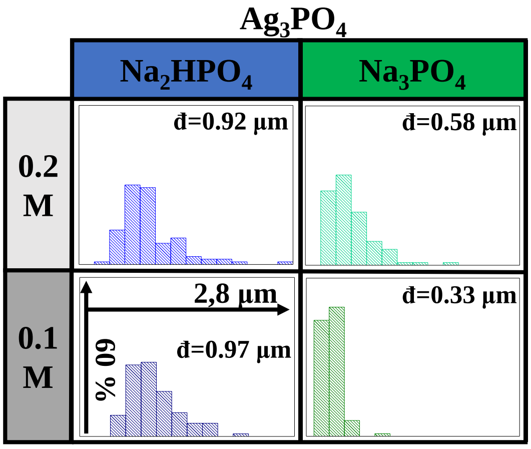

Figure S1. The particle size distribution of Ag_3PO_4 materials.

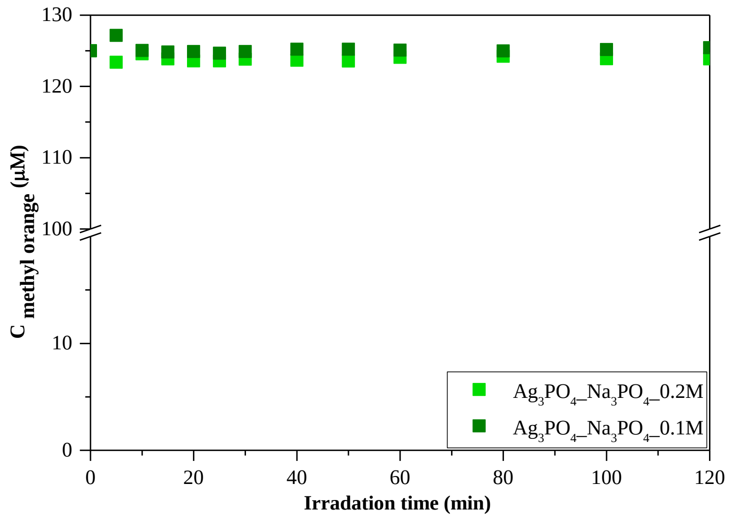

Figure S2. The adsorption test of the Ag_3PO_4_Na_3PO_4_0.2M and Ag_3PO_4_Na_3PO_4_0.1M on MO (C = 125 μM).
